# The Roles of Micronutrition and Nutraceuticals in Enhancing Wound Healing and Tissue Regeneration: A Systematic Review

**DOI:** 10.3390/molecules30173568

**Published:** 2025-08-31

**Authors:** Cristina Stanescu, Iulia Chiscop, Daniela Mihalache, Monica Boev, Camelia Tamas, Gabriela Stoleriu

**Affiliations:** 1 Department of Morphological and Functional Sciences, Faculty of Medicine and Pharmacy, “Dunarea de Jos” University, 35 Alexandru Ioan Cuza Street, 800216 Galati, Romania; daniela.mihalache@ugal.ro; 2 Clinical Surgical Department, Faculty of Medicine and Pharmacy, “Dunarea de Jos” University, 800008 Galati, Romania; iulia.chiscop@ugal.ro; 3 Department of Pharmaceutical Sciences, Faculty of Medicine and Pharmacy, “Dunarea de Jos” University, 35 Alexandru Ioan Cuza Street, 800010 Galati, Romania; monica.boev@ugal.ro; 4 Department of Plastic Surgery, Faculty of Medicine, “Grigore T. Popa” University of Medicine and Pharmacy, 16 Universitatii Street, 700115 Iasi, Romania; camelia.tamas@umfiasi.ro; 5 Clinical Medical Department, Faculty of Medicine and Pharmacy, “Dunarea de Jos” University, 35 Alexandru Ioan Cuza Street, 800216 Galati, Romania; gabriela.stoleriu@ugal.ro

**Keywords:** micronutrition, nutraceuticals, wound healing, antioxidants, polyphenols

## Abstract

Micronutrients and nutraceuticals play crucial roles in wound healing and tissue regeneration, supporting various physiological processes. This review aims to synthesize and evaluate the functions of various micronutrients and nutraceuticals, emphasizing the synergistic interactions among different nutrients that facilitate wound healing processes. A thorough literature review was performed using electronic databases, including PubMed, Scopus, Web of Science, Embase, Google Scholar, and Cochrane Library, to identify molecular studies, animal models, randomized controlled trials, and observational human studies published up to January 2000. Two independent reviewers screened the articles, extracted data, and evaluated the Risk of Bias using the Risk of Bias 2 (RoB 2) tool for the 190 studies that met the inclusion criteria. Evidence suggests that bioactive compounds found in functional foods and dietary supplements can help prevent chronic conditions and promote wellness beyond basic nutrition. Vitamins A, C, and E, as well as minerals such as zinc, selenium, and iron, are essential for cell proliferation and the formation of new tissues. Additionally, nutraceuticals, including omega-3 fatty acids, glutamine, arginine, and polyphenols, exhibit anti-inflammatory and antioxidant properties, which promote healing and reduce the risk of infection. Probiotics and other bioactive compounds in nutraceuticals contribute to maintaining the balance of microbiota, reducing inflammation, and stimulating cell regeneration. Significant variability was noted in study design, sample size, intervention dosage, and outcome measures. This evidence underscores the necessity for further well-designed clinical trials to determine the optimal dosages and combinations for specific wound types across diverse patient populations. This systematic review was prospectively registered in PROSPERO (ID: 1072091).

## 1. Introduction

Wound healing is a complex biological process that involves hemostasis, inflammation, proliferation, and tissue remodeling. Adequate nutritional support is essential for the efficient coordination of these phases. The metabolic demands of tissue repair increase the need for sufficient micronutrient intake, with deficiencies potentially delaying or impairing healing responses [1].

Micronutrition refers to the consumption of essential vitamins and minerals, which are crucial for maintaining health and supporting various physiological functions. The insufficient intake or deficiency of these micronutrients can significantly impact the wound healing process, leading to delayed healing, an increased risk of infection, and sub-optimal recovery outcomes [2]. Therefore, ensuring a proper diet that is rich in vitamins and minerals is vital for optimal wound healing, particularly in individuals at risk of deficiencies due to chronic diseases, malnutrition, or post-surgical recovery. In some cases, supplementation may be necessary to address specific deficiencies or meet increased nutritional needs [3]. Nutraceuticals are bioactive compounds that have demonstrated significant potential for supporting tissue regeneration and overall well-being, positively affecting the immune system, regulating physiological functions, and maintaining cellular integrity [4]. Deficiencies in micronutrients and nutraceuticals can result in sub-optimal cellular functions, the dysregulation of cytokine expression, compromised immune responses, and an increased vulnerability to inflammatory disorders and infections [5]. The optimal approach for nutritional supplementation to promote wound healing remains an area of active research.

Patients with chronic skin lesions are at an increased risk of developing depression and anxiety. When an individual experiences depression, various psychobiological changes can impede healing. For instance, depression can exacerbate stress, resulting in elevated levels of cortisol and other stress-related hormones, which have been associated with slower wound healing. Specifically, cortisol can interfere with the inflammatory phase of wound healing, delaying the subsequent proliferation and remodeling phases, which are necessary for optimal repair. Individuals experiencing depression frequently present diminished leukocyte activity and modified cytokine profiles, reducing the body’s capacity to effectively perform wound healing. This diminished immune function can lead to a slower and less efficient healing process. Holistic approaches that integrate both physical and psychological dimensions can significantly affect the treatment of skin disorders, enhance patient outcomes, and contribute to reduced stress levels. This stress reduction may, in turn, positively influence the immune system and facilitate the recovery process [6,7].

### 1.1. Objectives

This review aims to synthesize the findings from a range of studies to establish a comprehensive understanding of the targeted dietary interventions involving micronutrients and nutraceuticals that have the potential to improve wound healing outcomes and promote tissue regeneration. The synthesized evidence can potentially guide healthcare professionals in making informed decisions about nutritional interventions for wound healing. Through systematically examining the existing literature, this review highlights areas where further research is needed.

### 1.2. Vitamins and Their Role in Wound Healing

#### 1.2.1. Vitamin A

Vitamin A plays a crucial role in all stages of wound healing by stimulating epithelial growth, fibroblasts, granulation tissue, angiogenesis, collagen synthesis, epithelialization, and fibroplasia [8]. It stimulates epidermal renewal and increases the epithelialization rate during the inflammatory phase, and is involved in collagen production and scar formation during the maturation phase [9]. This is especially important for initiating the repair process and preparing the wound for the subsequent stages of healing. It also improves the production of extracellular matrix components such as type I collagen and fibronectin, increases the proliferation of keratinocytes and fibroblasts, and decreases the levels of matrix metalloproteinases [10]. Retinoids, which are derivatives of vitamin A, have the unique ability to reverse the inhibitory effects of anti-inflammatory steroids on wound healing. Anti-inflammatory corticosteroids are known to significantly impair wound healing, likely by reducing the levels of key growth factors such as transforming growth factor-beta (TGF-β) and insulin-like growth factor-I (IGF-I), as well as decreasing collagen production at the wound site. Retinoids counteract these effects by increasing the synthesis of TGF-β, IGF-I, and collagen, thereby improving the healing process [11]. The non-coding long RNA SNHG26 has been identified as a pivotal regulator of keratinocyte progenitors, which underpins the transition from the inflammatory to the proliferative phase. Mechanistically, SNHG26 interacts with nucleolin to modulate the expression of the c-Myc protein by enhancing its translation, thereby promoting cellular energy metabolism through hexokinase 2 [12].

Vitamin A deficiency can lead to various skin manifestations and affect the normal progression of wound healing, diminishing the ability of the skin to regenerate and repair itself due to impaired cell signaling and gene expression. This deficiency can lead to delayed wound closure, reduced dermal collagen deposition, and impaired epithelialization. Moreover, vitamin A deficiency can compromise the immune response, thereby increasing the susceptibility of wounds to infections [13]. Both local (topical) and systemic vitamin A supplementation have been shown to increase dermal collagen deposition. The potential benefits of vitamin A supplementation must be weighed against the associated risks, as vitamin A toxicity can be critical [14]. Therapeutic studies are needed to establish the efficacy and safety of vitamin A supplementation in the context of wound healing, and further research is needed to establish optimal dosage and safety guidelines.

#### 1.2.2. Vitamin C

Vitamin C is also involved in all phases of wound healing. It exhibits strong antioxidant properties and facilitates fibroblast activity, angiogenesis, and immune regulation during the inflammatory and proliferative phases [15]. The antioxidant properties of vitamin C make it a key player in protecting the immune system and reducing inflammatory responses. It promotes wound healing by favorably influencing the inflammatory, proliferative, and remodeling phases, as well as boosting extracellular matrix formation, which is essential for tissue regeneration [16]. In the inflammatory phase, it improves neutrophil migration and lymphocyte activation, and is necessary for apoptosis and neutrophil clearance [17]. During the proliferative phase, vitamin C contributes to the synthesis, maturation, secretion, and degradation of collagen, and plays a key role in the hydroxylation reactions required for collagen formation. These processes are vital in determining the structure and strength of new tissues formed at the wound site [18]. Vitamin C improves the production of extracellular matrix proteins, including type I collagen, as observed in studies on primary bovine osteoblasts. It also promotes fibroblast proliferation and induces the expression of self-renewal genes in neonatal human dermal fibroblasts [19,20]. Furthermore, vitamin C improves neutrophil migration and lymphocyte activation, and the topical application of vitamin C has been shown to accelerate fibroblast migration at certain concentrations. Vitamin C deficiency can adversely affect collagen production, thereby impairing scar formation and the overall maturation phase of healing [21].

#### 1.2.3. B-Complex Vitamins

B-complex vitamins play crucial roles in wound healing, particularly through their effects on the proliferation and migration of skin cells that are essential for tissue repair [22]; for example, they have been shown to have positive effects on human keratinocytes and fibroblasts during wound healing [23]. Vitamins B1 (thiamine), B2 (riboflavin), B3 (niacinamide), B5 (calcium d-pantothenate), B6 (pyridoxine HCl), B7 (biotin), B9 (folic acid), and B12 (cyanocobalamin) have been shown to positively influence wound healing [24]. These vitamins are essential for cellular metabolism, energy production, and wound healing. The successful completion of the wound healing cascade requires energy, amino acids, oxygen, metals, and trace minerals [25]. The interactions of B-complex vitamins with other nutrients are evident in the different phases of wound healing. In the inflammatory phase, B-complex vitamins act synergistically with vitamin A to enhance the release of cytokines, while their interactions with vitamin C improve neutrophil migration and lymphocyte activation. During the proliferative phase, vitamin B interacts with vitamin C to support collagen synthesis. In addition, zinc—which is necessary for DNA and protein synthesis and cell division—functions together with B vitamins to promote cell proliferation [26]. Combinations of certain B vitamins are more effective than individual vitamins; for instance, vitamins B1, B6, and B12 have jointly been associated with enhanced healing in burn wounds [27,28]. These findings underscore the potential benefits of incorporating B-complex vitamins into nutritional strategies aimed at optimizing the wound healing process. However, further research is required to establish evidence-based recommendations for their use in clinical settings.

#### 1.2.4. Vitamin D

Vitamin D plays a crucial role in the wound healing process, primarily through its impacts on epidermal and immune cell functions, such as improving the skin’s barrier function and promoting the production of antimicrobial peptides (e.g., cathelicidin and defensins) [29]. In this way, it helps to modulate the immune system, protect the skin from infection, and maintain its integrity. It facilitates the activation, migration, and re-epithelialization of stem cells from hair follicles and the epidermis, which are essential for wound closure and epidermal regeneration [30]. In addition, vitamin D influences the initial inflammatory response, which is beneficial for efficient wound closure. The anti-inflammatory effects of vitamin D protect against UV-induced damage [31]. In the context of tendon-to-bone healing—for example, after rotator cuff repair—vitamin D contributes to the proliferation and differentiation of osteoblasts, increases bone mineral density, and strengthens skeletal muscles. This process involves the liver-produced form of vitamin D, calcifediol, which enhances muscle cell function and metabolism [32,33,34]. Although the therapeutic potential of vitamin D in enhancing osteoblast differentiation and bone regeneration has been noted, determining the optimal administration route and dosage remains an open area of research [35]. Oral supplementation with high-dose vitamin D3 reduces the expression levels of pro-inflammatory mediators such as tumor necrosis factor α and inducible nitric oxide synthase. It also increases the expression of the anti-inflammatory mediator arginase-1 and genes related to skin barrier repair, thus helping to repair UV-induced DNA damage and reducing oxidative stress [36]. Low vitamin D levels are common among non-operatively treated adult patients with fractures, which can potentially impede fracture healing. Vitamin D deficiency has also been linked to a higher incidence of autoimmune and inflammatory conditions, which may further affect tissue regeneration and wound healing [37,38].

Vitamin D3 has been recognized as a principal regulator of cathelicidin expression in the skin. It functions by binding to vitamin D receptors, which subsequently dimerize with retinoid X receptors and attach to vitamin D response elements located in the promoter regions of vitamin D-responsive genes [39]. This process results in the increased production of cathelicidins, thereby enhancing the antimicrobial defense capacity of the skin [40]. This relationship underscores the potential of vitamin D-based therapies for the treatment of infectious and inflammatory skin diseases through the modulation of both innate and adaptive immune functions.

#### 1.2.5. Vitamin E

Vitamin E plays a multifaceted role in wound healing, primarily due to its antioxidant properties. It is known to influence cellular signaling and gene expression, as well as contributing to the modulation of inflammation, all of which are vital processes in wound management [41]. One review has highlighted that vitamin E can affect wounds infected with methicillin-resistant Staphylococcus aureus (MRSA), demonstrating its influence on wound healing through its antioxidant activity and effects on connective tissue growth factor (CTGF) [42]. Vitamin E has also shown positive effects on cell viability, proliferation, and migration, as well as reducing apoptosis in cells exposed to harmful substances such as nicotine [43]. Further studies have shown that formulations such as hyaluronic acid combined with vitamin E can improve mechanical properties in wound healing applications. Such formulations have been observed to promote better wound healing ability in vitro, indicating that the synergistic effects of vitamin E with other compounds might enhance their efficacy in wound management [44].

Research involving diabetic rats has demonstrated that palm vitamin E (PVE) and alpha-tocopherol enhanced the wound repairability and increased the activities of antioxidant enzymes, such as glutathione peroxidase and superoxide dismutase. These vitamin E preparations significantly reduced lipid peroxidation (demonstrated via lower malondialdehyde levels), indicating reduced oxidative stress, which is crucial for efficient wound healing [45]. Vitamin E metabolites have also shown promise in the context of wound healing. For instance, α-13′-carboxychromanol has been found to accelerate wound healing and improve the quality of newly formed tissues when applied topically. Its controlled release using bacterial nanocellulose in wound dressings offers potential as an advanced therapy for skin injuries, particularly under diabetic conditions [46].

Palm vitamin E (PVE), comprising a combination of tocotrienols and tocopherols, has been shown to exhibit superior efficacy in enhancing wound healing and promoting increased enzymatic free radical scavenging activities when compared with alpha-tocopherol alone. Tocotrienols are absorbed by cells more efficiently than alpha-tocopherol, and also exhibit superior antioxidant potency and anti-inflammatory effects [47]. These properties may contribute to a more effective wound healing process by mitigating inflammation, protecting cells from oxidative damage, and promoting tissue regeneration. Tocotrienols possess significant biological activities, such as neuroprotective and anticancer properties, which do not rely solely on their antioxidant capabilities [48,49]. Tocotrienols are often associated with triglyceride-rich lipoproteins, which may lead to their deposition in adipose tissue. The distinct lipoprotein associations of tocopherols and tocotrienols contribute to their different distributions in tissues and potentiate their biological functions. These differences in lipoprotein transport and tissue distribution may explain the unique roles of tocopherols and tocotrienols with respect to cellular functions, as well as their different effects on inflammation, cell proliferation, and apoptosis [50,51].

Tocopherols and tocotrienols appear to exhibit distinct patterns of plasma transport and tissue distribution. Tocopherols are mainly transported by low-density lipoproteins (LDLs) and high-density lipoproteins (HDLs). In contrast, tocotrienols exhibit different absorption pathways and tissue distributions: they are absorbed and cleared from plasma primarily through chylomicron clearance [52]. Tocotrienols are often associated with triglyceride-rich lipoproteins, which may lead to their deposition in adipose tissue. These distinct lipoprotein associations contribute to their different distributions in tissues and biological function [53], and may help to better explain their unique roles.

#### 1.2.6. Vitamin K

Vitamin K is predominantly recognized for its essential function in the coagulation cascade and, thus, its crucial role in the wound healing process [54]. When applied topically, it enhances wound contraction and decreases the overall healing duration. A randomized controlled trial has shown that the application of topical vitamin K significantly reduced the healing time of skin wounds when compared with a placebo group using Eucerin cream [55]. In addition to its well-documented role in coagulation, vitamin K is also involved in tissue renewal and cell growth, which are essential processes in wound healing. These activities are mediated by the activation of specific proteins that support endothelial integrity and tissue growth, which are crucial for repairing damaged tissues [56]. It also exhibits anti-inflammatory properties, offers protection against oxidative stress, and plays an important role in wound healing. Vitamin K plays a crucial role in reducing oxidative damage—especially in lipid-rich environments such as cell membranes—by modulating the expression levels of antioxidant enzymes and reducing reactive oxygen species (ROS). One significant function of vitamin K is the prevention of oxidative injury in neural cells—such as oligodendrocytes and neurons—which are highly vulnerable to oxidative stress [57]. In a study investigating oligodendrocyte development, it was observed that both vitamin K1 and menaquinone-4 (MK-4) at nanomolar concentrations effectively prevented arachidonic acid-induced oxidative toxicity by inhibiting 12-lipoxygenase [58]. This suggests that vitamin K may play a protective role in wound healing through the mitigation of oxidative stress in specific cellular contexts.

### 1.3. Essential Minerals Involved in Wound Healing

Minerals, such as zinc, calcium, magnesium, iron, and selenium, are also crucial for wound healing.

#### 1.3.1. Zinc

Zinc plays an important role in wound healing and acts as a cofactor for numerous metalloenzymes involved in cell membrane repair, cell proliferation, and immune system functions. It is essential for different stages of the wound healing process, including coagulation, inflammation, angiogenesis, the formation of new tissues, and remodeling of the extracellular matrix. Zinc serves as an essential cofactor in the functioning of cytoplasmic superoxide dismutase (Cu/Zn-SOD), an enzyme that mitigates cellular oxidative damage by facilitating the conversion of superoxide radicals into hydrogen peroxide and oxygen, the enzymatic activity of which is contingent upon the presence of both copper and zinc. Cu/Zn-SOD is a metalloenzyme in which copper is typically involved in the catalytic dismutation of superoxide anions while zinc plays a structural role, maintaining the conformation of the enzyme and enabling it to function properly [59].

Zinc also provides cytoprotection against reactive oxygen species (ROS) and bacterial toxins, helping to prevent cellular apoptosis. This antioxidant activity may be attributed to the influence of zinc on metallothioneins, which are cysteine-rich proteins that play protective roles in the cellular environment. This delicate balance is essential for proper wound healing, as an imbalance between active enzymes and their natural inhibitors can lead to the accelerated destruction of connective tissue [60,61].

While physiological concentrations of zinc (25 μM) did not significantly alter the morphology of epithelial cells, higher doses (250 μM) have been shown to induce cell rounding, loss of adhesion, and apoptotic characteristics. Furthermore, ZnO nanorods have been shown to induce angiogenesis by increasing the expression of VEGF, which plays a role in wound healing [62]. The utilization of zinc in wound management has been demonstrated to enhance healing rates, increase the expression of growth factors such as insulin-like growth factor-1 (IGF-1), and subsequently promote cell proliferation within granulation tissue, which is crucial in the wound healing process [63,64].

The topical administration of zinc stimulates wound epithelialization by improving the local defense system and collagenolytic activity [65]. MMP-8 and MMP-26 play important roles in keratinocyte migration during wound healing. MMP-8 (collagenase-2) is expressed during keratinocyte migration in both acute and chronic wounds, while MMP-26 exhibits a distinct expression pattern and is prominently expressed in the extracellular compartment of most chronic wounds, especially near the basement membrane area. In vitro experiments have demonstrated that MMP-26 inhibition results in keratinocyte migration and aberrant proliferation, indicating its crucial role in these processes. Notably, both MMP-8 and MMP-26 were expressed during keratinocyte migration in in vitro wound experiments, indicating a potential synergistic effect of these two MMPs in wound healing [66].

Zn deficiency can significantly delay wound healing. Studies in rats have shown that zinc-deficient subjects exhibit delayed healing of excised wounds and reduced tensile strength in incised wounds compared with controls, indicating the vital role of zinc in epithelialization and tissue integrity [67,68]. Excessive amounts of zinc can cause tissue damage and impair epithelial wound healing, highlighting the importance of proper dosing in zinc-based wound healing therapies.

#### 1.3.2. Selenium

Selenium plays crucial roles in wound healing due to its antioxidant, anti-inflammatory, and antimicrobial properties. It plays a vital role as a cofactor in the enzyme glutathione peroxidase (GPx), which is a significant part of the body’s antioxidant defense system. The active site of selenium-dependent glutathione peroxidase (GPx) comprises a selenium moiety—typically a single covalently bound selenium atom present in the form of the amino acid selenocysteine. This enzyme functions as a highly efficient catalyst for the reduction of various intracellular peroxides, including hydrogen and lipid peroxides, thereby detoxifying these potentially harmful molecules. Se-dependent GPx is vital for maintaining normal cellular biochemistry by protecting cells from oxidative damage [69]. Selenium is an essential immunonutrient which is present in the human body in the form of selenocysteine and selenoproteins, which act as antioxidants and indirectly participate in wound healing through the reduction in oxidative stress [70,71]. Selenium-stimulated exosomes have been demonstrated to significantly inhibit inflammation, enhance proangiogenic processes, and promote the proliferation and migration of human dermal fibroblasts [72]. Selenium nanoparticles (SeNPs) have shown impressive results in accelerating wound healing—particularly when combined with platelet-rich plasma (PRP)—in diabetic mouse models [73]. Nanocomposite hydrogels incorporating selenium nanoparticles have demonstrated excellent antibacterial, antioxidant, and anti-inflammatory capabilities, making them promising candidates for local applications. Selenium nanoparticles (SeNPs) have demonstrated significant potential for diverse biomedical applications due to their enhanced biocompatibility, bioavailability, and reduced toxicity compared with conventional selenium compounds [74].

Although selenium deficiency can lead to various health problems, excessive intake can also pose risks. The toxicology of inorganic nanoparticles, including SeNPs, is highly affected by various physicochemical parameters. Factors such as size, shape, surface properties, and synthesis narrow the therapeutic index of selenium, with toxic and therapeutic doses being relatively close together, raising concerns regarding its potential accumulation and long-term toxicity [75]. Therefore, further research is needed to determine the optimal dose of selenium for wound healing applications and to develop targeted delivery methods that maximize its therapeutic potential.

#### 1.3.3. Magnesium

Magnesium plays significant roles in wound healing, such as influencing various cellular processes and promoting tissue repair. Magnesium chloride (MgCl2) increases the expression of matrix metalloproteinase-7 (MMP7)—a cell migration-promoting factor—and enhances cell migration by activating the MEK/ERK pathway. This suggests that the application of Mg in the early stages of wound healing is beneficial. Magnesium silicate sprays (MSSs) exhibit improved degradability and excellent bioactivity for the promotion of cell proliferation and migration. An MSS was found to accelerate burn repair by regulating inflammation, improving vascularization, and stimulating collagen deposition without ectopic calcification [76]. Membranes incorporated within magnesium oxide (MgO) have a range of effects on wound healing processes; for example, they influence macrophage phenotype shifts to mitigate inflammatory responses, enhance fibroblast proliferation and collagen synthesis, and facilitate angiogenesis. In particular, these membranes stimulate macrophages to switch to a pro-healing M2 phenotype, stimulating TGF-β1 and reducing pro-inflammatory cytokines. This change in macrophage polarization helps to relieve inflammation and promotes a more favorable environment for wound healing [77]. The balance between M1 and M2 macrophages is crucial in various physiological and pathological processes. These mechanisms help to reduce inflammation and promote tissue repair, making Mg a promising element for the development of immunomodulatory biomaterials and wound healing strategies [78].

Magnesium intake is associated with lower levels of inflammatory markers such as C-reactive protein, leukocyte counts, and glycoprotein acetylation. Increased magnesium consumption is associated with a diminished risk of metabolic syndrome and cardiovascular disease, which has been attributed to enhanced insulin resistance, improved lipid profiles, and reductions in inflammation and oxidative stress [79,80]. Mg interacts with several nutrients to influence inflammation and metabolic health in complex ways. All enzymes involved in vitamin D metabolism require magnesium as a cofactor, emphasizing the importance of adequate magnesium intake to leverage the benefits of vitamin D, which regulates calcium and phosphate homeostasis to maintain bone health [81]. Mg deficiency can lead to inflammation through various mechanisms, including phagocyte cell activation, calcium channel opening, and NF-κB activation [82]. Overall, the interactions of Mg with other nutrients underscore its critical roles in various physiological functions and the potential implications of its deficiency or imbalance [83].

#### 1.3.4. Iron

Iron plays a crucial role in wound healing via various molecular and cellular mechanisms. One of the major components of wound healing is lactoferrin—an iron-binding glycoprotein that significantly contributes to skin repair by enhancing the initial inflammatory phase and balancing inflammation to prevent chronic wounds [84]. During the wound healing phase, iron is necessary for the synthesis of collagen, a vital extracellular matrix component that is essential for the remodeling phase. Collagen not only provides structural support but also acts as a substrate for new cell growth, promoting tissue repair and healing [85]. Furthermore, oxygen—another critical element in wound healing—requires adequate iron levels to effectively function. The supply of oxygen is crucial due to its involvement in reparative processes, such as cell proliferation, bacterial defense, and vascularization or angiogenesis [86]. Fe plays a supportive role in facilitating oxygen-dependent processes, enhancing wound closure, and maintaining tissue strength. Iron is also important in angiogenesis—a key process through which new blood vessels form from pre-existing vessels—thus providing nutrients and oxygen to the healing tissue. This creation of new vasculature is crucial for both delivering cells involved in tissue repair and clearing away metabolic waste products [87].

The release of iron into macrophages is essential for the repair and regeneration of local tissues, as demonstrated in studies using mice with the targeted inactivation of the iron exporter ferroportin. Ferroptosis is involved in various healing and regeneration processes, including inflammatory responses in skin wounds and the fibrosis of several organs. This suggests that a delicate balance of iron homeostasis is required during wound healing. Iron retention in macrophages due to ferroportin inactivation led to the delayed healing of skin wounds, the impairment of granulation tissue formation, and decreased fibroplasia. Iron retention affects stromal cell proliferation, blood and lymphatic vessel formation, and fibrogenesis, highlighting the importance of iron released from macrophages during wound healing [88].

Iron homeostasis and macrophage function are interconnected via ferroptosis—a form of regulated cell death characterized by iron overload and lipid peroxidation. In chronic wounds, such as those caused by diabetes, there is often persistent iron overload at the wound site due to recurrent infections and bleeding. Iron inhibits STAT1 activation and decreases the production of pro-inflammatory cytokines and inducible nitric oxide synthase (iNOS) by M1 macrophages. The NF-κB signaling pathway plays a crucial role in regulating iron metabolism in M1 macrophages [89]. Iron deficiency within macrophages results in the reduced proliferation of epithelial cells, the impaired growth of hair follicles, and delayed skin wound healing [90]. In summary, iron is integral to several mechanisms of wound healing, such as regulating inflammation through lactoferrin, synthesizing collagen, and supporting oxygen-dependent processes.

**Figure 1:**
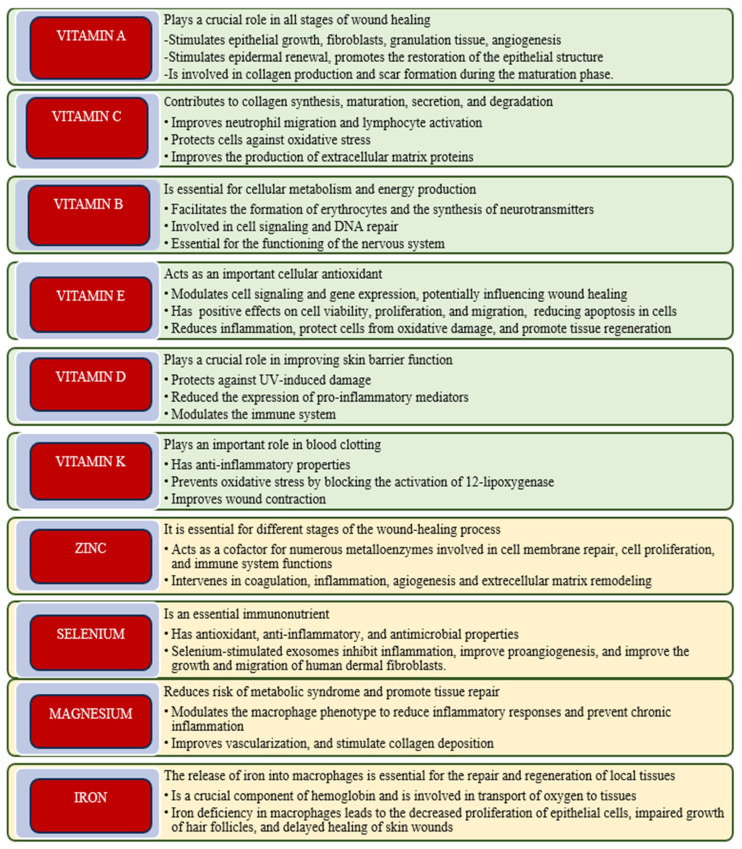
Roles of micronutrients in the wound healing process.

In contrast to macronutrients—such as proteins, carbohydrates, and fats—which are required in substantial quantities, micronutrients are essential in considerably smaller amounts. Nevertheless, they play vital roles in various physiological processes, including metabolic regulation, immune system function, cellular growth and repair, hormone production, enzyme activity, and antioxidant protection (Figure 1). Insufficient levels of these nutrients can compromise physiological functions and impede the body’s capacity to effectively regenerate tissues. Micronutrients can be acquired through a balanced diet that includes various fruits, vegetables, whole grains, lean proteins, and dairy products [91].

### 1.4. Probiotics Influence Skin Health and Wound Healing Processes

Probiotics have shown promising effects in the context of wound healing due to their antipathogenic, antibiofilm, and immunomodulatory properties [92]. They can competitively replace harmful bacteria, strengthen the epithelial barrier, and induce the migration and function of fibroblasts and epithelial cells [93]. This is particularly important considering the increasing antibiotic resistance of pathogens, and a balance between pathogenic and probiotic bacteria is crucial. Probiotics hold significant promise for wound management via various mechanisms; for example, they can modulate the wound microbiota, regulate immune responses, and facilitate tissue repair [94].

Several probiotic strains have shown promising results in improving wound healing processes, presenting varying degrees of effectiveness. *Lactobacillus bulgaricus* and *Lactobacillus plantarum* were shown to accelerate the healing of diabetic wounds in Wistar rats by modulating inflammatory cells and the expression of cytokines. *Lactobacillus bulgaricus* was effective in healing diabetic wounds, but showed no significant effect on wound repair in vitro [95,96]. *Bacillus subtilis natto* showed superior wound healing ability when compared with other strains, in a study using chitosan nanogels in Sprague Dawley rats. In addition, *Streptococcus thermophilus*, *Lactobacillus plantarum*, and *Lactobacillus acidophilus* were shown to promote re-epithelialization in a monolayer model of cells in vitro [97,98]. Probiotics influence the expression of cytokines in wound tissues by modulating the production of pro-inflammatory cytokines (e.g., TNF-α, IL-1β, and IL-6) while increasing the expression of anti-inflammatory cytokines (e.g., IL-10). These modulatory effects on cytokines help to regulate the inflammatory response during wound healing [99].

The mechanisms through which probiotics influence cytokine expression involve direct interactions with immune cells and indirect effects mediated through changes in the gut microbiota. Probiotics can activate intraepithelial lymphocytes, natural killer cells, and macrophages, thereby leading to cytokine-induced production [100]. In addition, probiotics can exert systemic effects through the gut–brain–skin axis, influencing skin health and wound healing processes. The gut microbiome, which is strongly influenced by probiotics, plays a pivotal role in modulating systemic inflammation and skin diseases, facilitating immune tolerance, and providing protection against pathogens [101]. Certain commensal bacteria, such as *Staphylococcus epidermidis*, can regulate gamma delta T-cells and induce the expression of perforin-2, which helps to eliminate harmful bacteria such as *Streptococcus aureus*, thereby facilitating wound healing [102,103]. Probiotics have been shown to positively influence skin health by promoting the proliferation of beneficial bacteria, suppressing pathogenic bacteria, and generating antimicrobial compounds [104]. They can boost the immune system, improve the levels of skin barrier components, and modulate skin inflammation. Probiotics are effective in improving conditions such as atopic eczema and atopic dermatitis, as well as in improving the innate immunity of the skin [105]. Oral probiotics represent a promising, straightforward, safe, and cost-effective strategy for managing skin inflammation [106]. However, further research is necessary to fully elucidate its therapeutic potential in the field of dermatology.

### 1.5. Effects of Omega-3 Fatty Acids on Wound Healing

#### 1.5.1. Omega-3 Fatty Acids

Omega-3 fatty acids are integral to wound healing because of their immunomodulatory properties and capacity to facilitate tissue repair. Linoleic and alpha-linolenic acids are the only fatty acids deemed essential for human health. However, under specific conditions, such as chronic inflammation, pregnancy, advanced age, and certain dermatological disorders, eicosapentaenoic acid (EPA), docosahexaenoic acid (DHA), and gamma-linolenic acid (GLA) may also become essential. These essential fatty acids serve as precursors to lipid mediators of inflammation, which play crucial roles in modulating the inflammatory response during wound healing [107]. Resolvin E1 (RvE1)—a specialized mediator derived from omega-3 fatty acids—has been shown to promote intestinal wound repair. RvE1 is produced locally in response to intestinal mucosal damage and improves wound healing by increasing cell proliferation and migration through the activation of various signaling pathways, including those involving CREB, mTOR, and Src-FAK. Furthermore, RvE1 induces the activation of the small GTPase Rac1, resulting in the enhanced production of intracellular reactive oxygen species and increased cell–matrix adhesion. Notably, the increased consumption of omega-3 fatty acids can stimulate the endogenous production of Resolvin E1 [108].

Omega-3 fatty acids also modulate inflammatory pathways by influencing T-cell differentiation and gene expression. They affect the membrane composition, eicosanoid biosynthesis, cell signaling cascades, and gene expression in almost every cell in the body. In the field of wound healing, supplementation with eicosapentaenoic acid (EPA) has been correlated with elevated concentrations of interleukin-10 (IL-10) in tissues during the early phases of the healing process. Omega-3 fatty acids have been shown to activate high-conductance voltage-activated (BK) calcium (Ca^2+^) and potassium (K^+^) channels, which contribute to vasodilation. This activation occurs through the destabilization of the closed conformation of the ion conduction gate, potentially affecting the flow of blood to the wound site [109].

Research has indicated that a combination of arginine, glutamine, omega-3 fatty acids, vitamins, and trace elements can improve wound healing and reduce infectious complications. Arginine has been identified as an essential nutrient that is crucial in certain clinical situations, such as in trauma patients or those at high risk of malnutrition. When combined with omega-3 fatty acids, arginine appeared to have a synergistic effect, producing more significant benefits than either nutrient alone. This combination has been shown to have positive effects on wound healing, infection, and other inflammatory conditions [110].

The anti-inflammatory properties of omega-3 fatty acids are mediated by several pathways. For example, they inhibit the formation of pro-inflammatory eicosanoids derived from omega-6 fatty acids such as prostaglandin E2 (PGE2) and leukotriene B4 (LTB4). In addition, omega-3 fatty acids serve as precursors for the synthesis of specialized pro-resolution mediators (SPMs)—including resolvins, protectins, and maresins—which actively promote the resolution of inflammation. Animal studies have consistently shown the strong anti-inflammatory and immunomodulatory effects of omega-3 fatty acids in various diseases, including those related to autoimmunity, arthritis, and infection [109].

Food sources of omega-3 and omega-6 fatty acids have distinct impacts on health. Omega-3 fatty acids—especially EPA and DHA, which are found in fish oil and marine sources—have strong anti-inflammatory properties and suppress pro-inflammatory cytokines, such as IL-1β, TNF-α, and IL-6. These fatty acids play crucial roles in brain development, cardiovascular health, and the prevention of chronic diseases. In contrast, omega-6 fatty acids tend to be pro-inflammatory and can promote the pathogenesis of many chronic diseases, including cardiovascular diseases. A high dietary omega-6/omega-3 ratio is associated with increased risks of obesity, coronary heart disease, hypertension, cancer, diabetes, and autoimmune diseases [111].

Genetic factors play a significant role in influencing individual responses to dietary omega-6/omega-3 ratio adjustment. Individuals possessing genetic variants of the 5-lipoxygenase (5-LO) gene exhibit differential responses to dietary omega-6 and omega-3 fatty acids. An increase in dietary arachidonic acid (AA) enhances the atherogenic effect associated with this genotype, whereas an elevated intake of omega-3 fatty acids—specifically eicosapentaenoic acid (EPA) and docosahexaenoic acid (DHA) —mitigates this effect [112].

Recent studies have discovered a new mechanism underlying the anti-inflammatory effect of omega-3 fatty acids on the gut microbiota. Omega-3 fatty acids improve the production and secretion of intestinal alkaline phosphatase, which induces changes in the composition of the intestinal bacteria. This leads to decreased lipopolysaccharide production and intestinal permeability, ultimately reducing metabolic endotoxemia and inflammation [113]. Omega-3 supplementation has the potential to inhibit inflammatory pathways, including NLRP3, TLR4, and NOD2 inflammasome signaling. This inhibition may lead to the suppression of the NF-κB pathway and a subsequent reduction in the secretion of pro-inflammatory cytokines [114].

Food sources of omega-3 fatty acids and supplements can have different effects on the gut microbiota, potentially influencing overall health and athletic performance. Natural food sources of omega-3s—such as fatty fish, nuts, and seeds—provide a more complex nutritional profile that can positively influence the gut microbiota. These whole foods contain not only omega-3s but also other nutrients such as fiber, which can act as prebiotics and support beneficial gut bacteria [115].

#### 1.5.2. Arginine and Glutamine

Arginine and glutamine play crucial roles in improving collagen deposition during wound healing via various mechanisms. Their availability determines the quality and speed of collagen matrix assembly during the proliferative and remodeling phases. Arginine serves as the sole substrate for the synthesis of nitric oxide (NO), which is vital for wound healing. NO has been shown to improve collagen synthesis in human tendon cells in vitro, potentially explaining the beneficial effects of NO donors in animal models and clinical trials of tendinopathies. In addition, arginine can be metabolized to arginase-1 ornithine, which leads to the production of l-proline—a substrate for collagen synthesis [116,117]. Although low doses of exogenous NO improve total protein and collagen synthesis in tendon cells, high doses can inhibit collagen synthesis. This suggests that the effects of arginine on collagen deposition may be dose-dependent. Both arginine and glutamine are considered important nutrients for wound healing, and arginine supplementation shows promise for improvements in both acute and chronic wound healing [118,119].

Amino acids play integral roles in wound healing by supporting cellular energy production, collagen synthesis, and immune responses. Although they can reduce inflammation, excessive anti-inflammatory action can affect the healing process [120]. Amino acids—especially arginine and glutamine—are considered to be important for wound healing. Arginine is conditionally essential in certain clinical situations, such as in trauma patients. Protein loss can negatively affect the entire immune process involved in wound healing. Arginine and glutamine play crucial roles in modulating immune responses during wound healing, acting as “immunonutrients” that have significant impacts on various aspects of the immune system and tissue repair processes [121,122]. Arginine is an essential amino acid that is particularly important for wound healing. It has been shown to improve wound resistance and collagen deposition in incisional wounds in both rodents and humans. Arginine is involved in protein synthesis, cell signaling (through nitric oxide production), and cell proliferation (through its metabolism to ornithine and polyamines), rendering arginine an essential substrate for wound healing [123,124].

### 1.6. The Effectiveness of Polyphenols in Wound Healing

Polyphenols represent a broad category of compounds distinguished by the presence of multiple phenol units, as reflected in the term “polyphenols.” These compounds can be categorized into two primary groups: flavonoids and non-flavonoids. Non-flavonoid polyphenols include phenolic acids, stilbenes, lignans, and tannins. This extensive classification encompasses a diverse array of compounds with varying levels of complexity and physiological functions.

Polyphenols have demonstrated significant potential for promoting wound healing through several mechanisms. They have been shown to improve healing times, reduce infection rates, and improve tissue regeneration in clinical trials as well as in vivo and in vitro studies. While polyphenols generally facilitate angiogenesis, the wound healing process encompasses both pro- and anti-angiogenic phases. Initially, there is the excessive formation of blood vessels to rapidly restore blood flow; this is followed by an anti-angiogenic phase, during which the vascular network matures and the number of vessels is reduced. The ability of polyphenols to modulate this balance may contribute to their observed efficacy in promoting wound healing [125].

Oxidative stress significantly influences the effectiveness of polyphenols in wound healing, with polyphenols showing particular promise in addressing the challenges posed by the excessive production of reactive oxygen species (ROS). Excessive oxidative stress can lead to impaired wound healing, especially in patients with chronic wounds and diabetes [126]. Polyphenols have been demonstrated to promote wound healing in environments with high oxidative stress. In particular, they possess the ability to improve antioxidant defenses, reduce oxidative stress, modulate inflammatory responses, improve the healing time, reduce infection rates, and improve tissue regeneration [127].

However, their poor stability in the gastrointestinal tract and low bioavailability limit their practical application. Nanotechnology provides a solution to these challenges, improving the stability, bioavailability, and targeted delivery of polyphenols. Nanoencapsulation techniques protect polyphenols from degradation in the gastrointestinal tract and maintain their original characteristics during processing, storage, and digestion. Various nanocarriers—such as nanoparticles, nanoemulsions, nanomyceles, and nanolipids—have been developed to improve the physicochemical properties of polyphenols [128]. Liposomes have been widely used as drug delivery systems for wound healing. Polymeric nanoparticles, liposomes, and certain inorganic nanoparticles appear to be particularly effective for delivering polyphenols in wound healing applications. The choice of nanoparticle type often depends on the specific polyphenol delivered and the targeted wound healing application. The properties of polymer nanoparticles, such as their hydrophobicity, size, charge, and surface changes, play crucial roles in their ability to overcome biological barriers and effectively distribute their cargoes in target tissues. These characteristics can be leveraged to improve the delivery of specific polyphenols for wound healing, making them promising candidates for use as active delivery systems in wound management [129].

**Figure 2:**
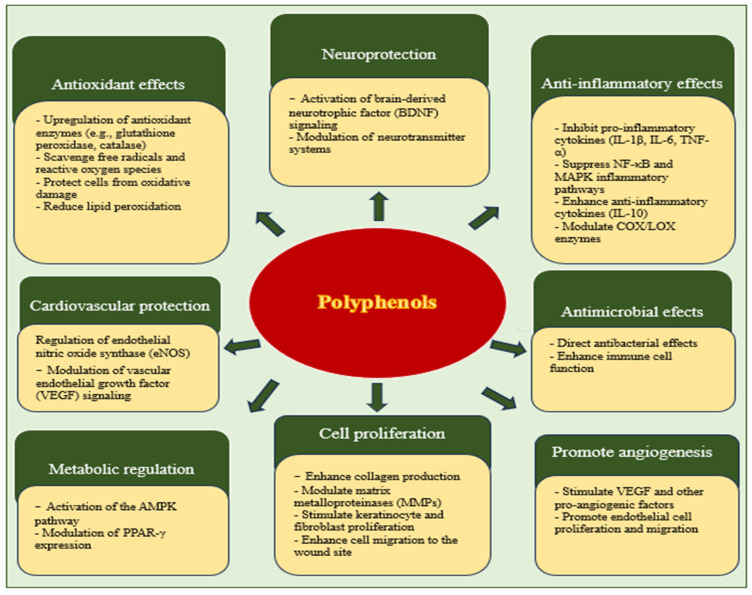
The beneficial effects of polyphenols in the context of wound healing.

The effectiveness of polyphenols in wound healing does not depend solely on their antioxidant properties. They also exhibit anti-inflammatory and antimicrobial properties, which are crucial for the management of hard-to-heal wounds, especially in diabetic and elderly populations [130]. How polyphenols significantly promote wound healing is further detailed in Figure 2.

#### 1.6.1. Resveratrol

Resveratrol—a natural polyphenol—plays a significant role in wound healing due to its various biological effects. It possesses powerful anti-inflammatory, antioxidant, and cellular protective properties that contribute to the acceleration of wound healing and tissue regeneration. Resveratrol promotes wound healing by improving cell proliferation, reducing apoptosis, and promoting epidermal and dermal repair, collagen synthesis, and scar maturation [131]. It activates various pathways and interacts with numerous substances to protect the skin against harmful UV radiation and improve collagen synthesis [132]. Additionally, resveratrol promotes angiogenesis by activating VEGF, which is crucial for tissue regeneration. It regulates genes associated with extracellular matrix components and modulates inflammatory and immunological pathways, including cytokine–cytokine receptor interactions, chemokine signaling, and tumor necrosis factor (TNF) signaling. Resveratrol activates the estrogen receptor, leading to increased collagen production [133]. This compound also accelerates the formation of granulation tissue and collagen deposition, thereby facilitating wound healing. In keloid environments characterized by excessive collagen production, resveratrol decreases the expression of type I collagen in keloid fibroblasts without negatively affecting normal skin fibroblasts. This suggests that resveratrol may have an anti-fibrogenic effect, helping to prevent excessive scarring [134,135].

Resveratrol inhibits the MAP signaling cascade, specifically through inhibiting the phosphorylation of extracellular signal-regulated kinase (ERK) and p38 MAPK. This inhibition of MAPK pathways by resveratrol leads to the decreased activation of downstream transcription factors and the reduced expression of pro-inflammatory mediators. In collagen-induced arthritis, resveratrol decreased the mRNA expression of MAPK3, suggesting a direct effect on the MAPK pathway involved in inflammatory arthritis [136]. Resveratrol has been shown to inhibit TGF-β signaling, which plays a crucial role in regulating cellular processes such as proliferation, migration, and angiogenesis. Resveratrol suppresses fibrosis and inflammation by suppressing TGF-β, thereby affecting the production and degradation of collagen [137].

Its ability to modulate inflammation, oxidative stress, autophagy, collagen proliferation, and angiogenesis makes it a versatile compound for skin regeneration. In addition, resveratrol-laden biomaterials such as hydrogels and nanoparticles have been shown to possess improved wound healing properties, suggesting potential applications in tissue engineering and wound dressings [138].

#### 1.6.2. Curcumin

Curcumin—an active constituent of turmeric (*Curcuma longa* L.) —is a promising natural compound with antioxidant, antimicrobial, and anti-inflammatory properties. These attributes are essential for the wound healing process, which encompasses coagulation, inflammation, tissue formation, and remodeling [139]. The antioxidant properties of curcumin facilitate the scavenging of free radicals and reduce oxidative stress, which is vital for protecting tissues during the inflammatory phase of wound healing [140]. This reduction in oxidative stress also aids in the prevention of chronic inflammation, thereby creating an environment which is conducive to collagen synthesis and tissue repair. Furthermore, curcumin increases the activities of antioxidant enzymes such as superoxide dismutase, catalase, and glutathione peroxidase, which further contributes to its protective effect against cellular damage [141].

The role of curcumin as an anti-inflammatory agent involves the downregulation of pro-inflammatory cytokines, such as tumor necrosis factor alpha (TNF-α) and interleukin-1 (IL-1). Through shortening the inflammatory phase and preventing chronic inflammation, curcumin allows for a quicker transition to the proliferative phase of wound healing, in which collagen synthesis is predominant. Furthermore, curcumin facilitates the migration, proliferation, and differentiation of fibroblasts, which are crucial steps in tissue remodeling and wound closure [142]. Research has indicated that curcumin enhances the expression of transforming growth factor-beta1 (TGF-beta1)—a critical factor in wound healing processes. TGF-beta1 facilitates wound repair by promoting cellular proliferation, collagen deposition, and angiogenesis. These processes are essential for the formation of new tissues and restoration of the skin barrier, thereby promoting tissue regeneration and repair [139]. Curcumin not only promotes the synthesis of collagen, but also aids in its maturation and cross-linking. This leads to increased tensile strength and the better organization of collagen fibrils within the wound area, which are crucial for the formation of strong and resilient scar tissue. Curcumin treatment improves wound maturation by affecting the physiological environment and increasing the stability of acid-soluble collagen [143].

Although curcumin demonstrates significant potential for wound healing, its practical application is constrained by its limited solubility and rapid metabolism. Recent technological advancements such as the development of curcumin-loaded nanostructured lipid carriers have improved its bioavailability. These innovative carriers significantly improve the wound healing efficacy of curcumin, promoting faster wound closure and enhancing antimicrobial activity [144]. The incorporation of curcumin into nanocarriers such as liposomes and nanoparticles can further enhance its bioavailability and efficacy in promoting collagen synthesis. These formulations allow for the targeted and sustained release of curcumin at the wound site, increasing its therapeutic effects and promoting more effective wound healing [145,146,147].

### 1.7. Flavonoids

Flavonoids are polyphenolic compounds which are commonly found in fruits, vegetables, and other plant-based sources. The significant feature of flavonoids is their wide structural diversity and extensive presence throughout the plant kingdom. They consist of two aromatic rings connected by three carbon atoms, forming a closed pyran ring. This structural foundation leads to various subclasses, such as flavonols, flavones, flavanones, isoflavones, catechins, anthocyanidins, and chalcones, each differing in their specific chemical substitutions. Flavonoids are known for their antioxidant, anti-inflammatory, and antimicrobial properties, leading to certain effects on wound healing. Flavonoids act on the wound healing process by modulating the expression of biomarkers in several pathways, including the Wnt/β-catenin, Hippo, TGF-β, Hedgehog, JNK, Nrf2/ARE, NF-κB, MAPK/ERK, Ras/Raf/MEK/ERK, PI3K/Akt, and NO pathways. Specifically, flavonoids can eliminate reactive oxygen species, enhance endogenous antioxidants, reduce the expression and synthesis of inflammatory cytokines (IL-1β, IL-6, TNF-α, and NF-κB), inhibit inflammatory enzymes, improve anti-inflammatory cytokines (IL-10), and control blood sugar levels. They also regulate matrix metalloproteinases and stimulate angiogenesis and extracellular matrix formation [148].

Flavonoids exhibit powerful antioxidant properties that significantly contribute to the wound healing process. These compounds act as free radical scavengers and metal chelators, thus reducing oxidative stress, which is crucial for promoting the repair and regeneration of tissue. Their antioxidant activities help to protect cells against oxidative damage, thus supporting the healing process. Flavonoids can also chelate metal ions involved in oxidative reactions [149].

Flavonoids inhibit lipid peroxidation during wound healing by directly removing reactive oxygen species (ROS), enhancing endogenous antioxidants, chelating metal ions, and synergistically interacting with other antioxidants. These mechanisms contribute to their potential as therapeutic agents for wound management, especially in diabetic conditions (where oxidative stress is typically high) [150]. Flavonoids directly increase the levels of enzymatic and non-enzymatic antioxidants in newly formed tissues. In diabetic wounds, flavonoids such as quercetin, hesperidin, and curcumin effectively eliminate ROS, enhance endogenous antioxidants, and reduce the expression and synthesis of inflammatory cytokines. They can also improve the production of the anti-inflammatory cytokine IL-10, which further aids in wound healing [151].

The regulation of MMP expression by flavonoids appears to involve complex interactions among different MAPK pathways; for example, in oral keratinocytes, calcium-induced MMP-9 expression is differentially regulated by the ERK1/2 and p38 MAPK pathways, with p38 MAPK activity downregulating this process. Some flavonoids—such as quercetin—inhibit the activation of both ERK and p38 MAPK, while kaempferol inhibits the activation of p38 MAPK and JNK [152].

#### 1.7.1. Proanthocyanidins

Proanthocyanidins—a category of bioactive flavonoids—are essential in facilitating wound healing. These compounds are abundant in various wild berries and have long been used in traditional medicine to treat skin wounds. Research into the wound healing properties of proanthocyanidins has highlighted their capacity to influence multiple phases of the healing process through several mechanisms. Proanthocyanidins enhance the closure of scratch wounds by stimulating mitochondrial bioenergetics, including increasing basal respiration, ATP production, and maximum respiratory capacity. This metabolic enhancement involves cellular proliferation and migration, and is crucial during the early stages of wound healing [153].

Furthermore, proanthocyanidins upregulate the expression of key extracellular matrix (ECM) proteins such as integrin-ß1 and collagen type I α2 chains. This upregulation is pivotal for supporting the structural framework necessary for tissue repair and regeneration. Through targeting cellular bioenergetics and integrin-mediated cell–ECM signaling, proanthocyanidins contribute to the acceleration of wound healing processes and offer potential therapeutic benefits for chronic wounds and inflammatory skin disorders [154].

The antioxidant and anti-inflammatory properties of proanthocyanidins also play crucial roles in wound healing. They help to mitigate oxidative stress by scavenging reactive oxygen species (ROS), thereby reducing cellular damage and providing a conducive environment for healing. The suppression of inflammation further aids in faster wound closure by stabilizing the local wound environment, which is essential for the proliferation and migration phases of wound healing [155]. When compared to other polyphenols such as resveratrol, proanthocyanidins exhibit certain similarities and differences. While both proanthocyanidins and other polyphenols exhibit antioxidant and antimicrobial properties that are crucial for wound healing, their specific actions and effects on the expression of associated genes might differ. Proanthocyanidins are known for their contribution to cardiovascular health, particularly through their capacity to modulate inflammatory responses and lipid levels. These effects can indirectly enhance wound healing by improving the physiological conditions that promote tissue repair [156,157].

Each type of polyphenol—including proanthocyanidins—may offer unique pathways and benefits in the wound healing process; thus, ongoing research is crucial for the optimization of their use in clinical applications.

#### 1.7.2. Quercetin

Quercetin—a naturally occurring flavonoid found in many fruits and vegetables—has shown promising wound healing properties. Its efficacy is largely attributed to its antioxidant and anti-inflammatory activities, which significantly contribute to the healing process. Moreover, quercetin is effective in addressing specific wound conditions, such as pressure ulcers. In cases of ischemia–reperfusion-induced lesions, quercetin treatment significantly reduced immune cell infiltration and pro-inflammatory cytokine production, further highlighting its potential as a therapeutic agent for such conditions [158]. Quercetin functions as an antioxidant and reduces oxidative stress at wound sites, which can hinder healing. It also exerts anti-inflammatory effects by modulating cytokine expression, such as increasing interleukin-10 (IL-10) and decreasing tumor necrosis factor-alpha (TNF-α), leading to an improved inflammatory response during the healing process [159].

Quercetin enhances the expression of key growth factors such as vascular endothelial growth factor (VEGF) and transforming growth factor-beta1 (TGF-β1), which play crucial roles in angiogenesis and tissue remodeling—essential processes for effective wound healing. Quercetin’s ability to modulate these factors leads to the proliferation of fibroblasts and encourages cell migration, contributing to efficient wound closure. Quercetin promotes cell motility by interacting with the cytoskeleton [160]; for instance, its effect on the ATP-binding cassette transporter ABCC6 has been linked to cytoskeletal rearrangements that facilitate HepG2 cell motility. Quercetin affects several intracellular signaling pathways that regulate cell migration. It has been shown to modulate the MAPK pathway, which involves the activation of ERK and p38 kinases that are critical for cell migration and wound healing processes [161].

Studies have shown that quercetin—especially when used in combination with other compounds such as curcuminoids—enhances cell migration and wound healing more effectively. These combinations leverage synergistic effects to enhance antimicrobial, antioxidant, and cell migratory activities, thereby promoting efficient wound healing [162]. The topical application of quercetin has been observed to improve wound repair and regeneration, particularly under diabetic conditions, mediated by the modulation of important cytokines and growth factors [163]. One effective form of quercetin application is through the use of innovative delivery systems such as bimetallic nanoparticles and hydrogels. Quercetin-functionalized nanoparticles have demonstrated promising binding potential with critical targets in wound healing, leading to enhanced wound closure. Similarly, quercetin nanocrystal-loaded alginate hydrogel patches have been developed, which allow for the sustained release of quercetin to reduce oxidative stress and accelerate wound repair [164]. Quercetin’s capacity to modulate essential biological pathways provides a compelling rationale for its incorporation into therapeutic strategies for chronic wound management.

#### 1.7.3. Fisetin

Fisetin—a flavonoid found in plants—has been shown to effectively suppress UVA-induced MMP-1 and MMP-3 expression in human dermal fibroblasts and epidermal keratinocytes, achieved through the inhibition of the NOX/ROS/MAPK pathway. These findings suggest that the specific structural characteristics of these flavonoids contribute to their ability to modulate MMP expression [165]. The number and arrangement of hydroxyl groups in flavonoids play crucial roles in their MMP inhibitory activities. Flavonoids with a higher number of hydroxyl groups—especially on the B-ring—demonstrate stronger antioxidant properties and the more effective inhibition of MMP expression [166].

The absorption of flavonoids is significantly influenced by dietary lipids, and their structural properties play crucial roles in this process. According to a previous study, soybean oil digestion products (SOEDs) increase the apparent permeability coefficients of most dietary flavonoids [167]. The interactions between lipids and flavonoids during absorption can be significantly influenced by the presence of other nutrients, particularly in terms of their effects on transport proteins and membrane properties. Dietary nutrients have been found to modulate the expression or function of transporter proteins, consequently synergistically or antagonistically affecting flavonoid absorption [168].

### 1.8. Saponins

Saponins are bioactive phytochemical compounds present in natural products that offer a range of health benefits for humans, including hypocholesterolemic, immunoadjuvant, anti-inflammatory, and neuroprotective effects. They facilitate wound healing due to their medicinal properties [169]. Natural products containing saponins, essential oils, flavonoids, and phenolic compounds have been shown to have potential for use in wound healing applications due to their anti-inflammatory, antioxidant, antibacterial, and pro-collagen synthesis properties [170]. These properties help to reduce inflammation, protect against oxidative stress, and prevent infections at the wound site. In addition, saponins have been shown to stimulate collagen synthesis and promote cell proliferation, which are essential for tissue regeneration and wound closure. Saponins exert anti-inflammatory effects by regulating the NF-κB and MAPK pathways, as well as inhibiting COX/LOX enzymes [171]. They also influence the composition and activity of the gut microbiome, contributing to their anti-inflammatory effects [172].

Table 1 provides a summary of the roles of nutraceuticals in wound healing and their underlying mechanisms.

**Figure 3:**
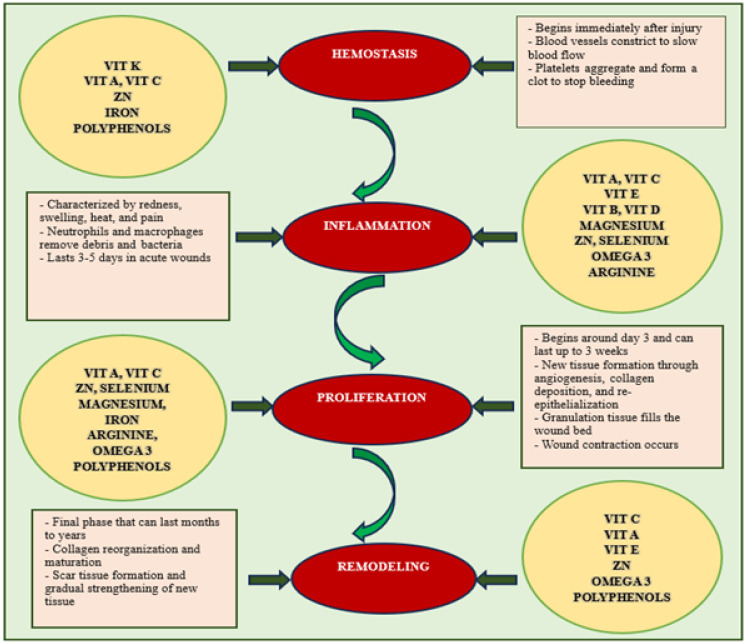
Micronutrients and nutraceuticals in different phases of wound healing.

Micronutrients and nutraceuticals play crucial roles in the various phases of wound healing, working synergistically to promote tissue repair (see Figure 3). Proper nutrition, oxygenation, and wound care are crucial for ensuring optimal progression through these stages.

## 2. Methods and Materials

### 2.1. Eligibility Criteria

**Inclusion and Exclusion Criteria**

Studies were selected for inclusion if they met the following predefined criteria.

**Inclusion Criteria:**

- Peer-reviewed original research, clinical trials, and reviews examining the impacts of nutraceuticals on wound healing and tissue regeneration.
- Previous studies that focused on the effects of vitamins, minerals, and amino acids on wound healing and tissue regeneration.
- Articles written in English and available in full-text format.
- Human and animal model studies providing mechanistic or clinical insights.

**Exclusion Criteria:**

- Publications in languages other than English.
- Editorials, opinion pieces, or conference abstracts without full data.
- Studies lacking specific outcome measures related to wound healing or tissue regeneration.
- Research published before 2000.

### 2.2. Information Sources and Search Strategies

This narrative review was conducted following the PRISMA 2020 guidelines for the systematic synthesis of qualitative evidence, adapted for a thematic and integrative approach. A comprehensive literature search was performed across six major electronic databases: PubMed, Scopus, Web of Science, Embase, Google Scholar, and Cochrane Library. The search spanned publications between January 2000 and April 2025.

The following Medical Subject Headings (MeSH) terms and free-text keywords were used in various combinations: “micronutrition”, “nutraceuticals”, “wound healing”, “tissue regeneration”, “angiogenesis”, “collagen synthesis”, and “bioavailability”. Relevant articles were screened based on their titles and abstracts, and full texts were reviewed for inclusion. Manual cross-referencing of the bibliographies of the included articles was also conducted to identify additional eligible sources.

### 2.3. Selection Process

Two independent researchers (C.S. and G.S.) conducted a screening of the titles and abstracts of the studies, excluding duplicates and those deemed irrelevant. Full-text articles were obtained for studies that passed this initial screening. The same two authors then independently assessed these articles for adherence to the eligibility criteria. Any disagreements during the screening process were resolved through discussion.

### 2.4. Data Collection Process and Data Items

All retrieved articles were screened independently by the two reviewers based on their titles and abstracts, followed by full-text evaluation for eligibility. Discrepancies in the inclusion decisions were resolved through discussion and consensus. After applying these criteria, 190 articles were included in the final synthesis.

### 2.5. Study Risk of Bias Assessment

Quality assessment was conducted utilizing the Risk of Bias 2 (RoB 2) tool, as outlined in the *Cochrane Handbook for Systematic Reviews of Interventions*. While the RoB 2 tool offers a comprehensive and structured framework for evaluating the Risk of Bias in randomized trials, its effective implementation necessitates meticulous planning and adequate resources. This tool assesses the Risk Of Bias across five domains: (1) bias arising from the randomization process; (2) bias due to deviations from the intended interventions; (3) bias resulting from missing outcome data; (4) bias from the method of measuring the outcome; (5) bias in the selection of the reported result. Two authors (C.S. and G.S.) independently conducted the assessment, categorizing each domain as either “Low,” “Some Concerns,” or “High.” Studies were deemed to have an overall high Risk of Bias if one or more domains received a “High” rating, or if more than two domains were rated as having “Some Concerns.” Additionally, studies with two domains rated as having “Some Concerns” were considered to have some concerns for bias overall. Any disagreements were resolved through discussion.

### 2.6. Effect Measures and Statistical Analysis

Key variables such as nutrient type, dosage, model system (in vitro, in vivo, or clinical), measured biomarkers (e.g., inflammatory cytokines, MMP activity, and angiogenesis indicators), and primary outcomes were extracted. A thematic synthesis approach was employed to categorize the retrieved findings based on nutrient class and mechanistic domain (e.g., antioxidant activity, immune modulation, and collagen formation). Where applicable, emphasis was placed on studies reporting translational or clinical outcomes, including randomized controlled trials and interventional studies. The narrative structure was designed to highlight inter-nutrient synergy, emerging delivery strategies, and areas that require further research.

This review presents evidence from molecular studies, animal models, randomized controlled trials, and observational human studies to offer a comprehensive overview of micronutrient and nutraceutical interventions focused on wound healing and tissue regeneration.

The primary outcome of this systematic review is wound healing efficacy, measured in terms of factors such as the wound closure rate, tissue regeneration, inflammation reduction, infection rate, and collagen synthesis. The secondary outcomes of this systematic review include the bioavailability and pharmacokinetics of nutraceutical compounds, adverse effects or toxicity reported in preclinical or clinical studies, pain management, and patient quality of life assessment.

### 2.7. Study Selection

**Figure 4:**
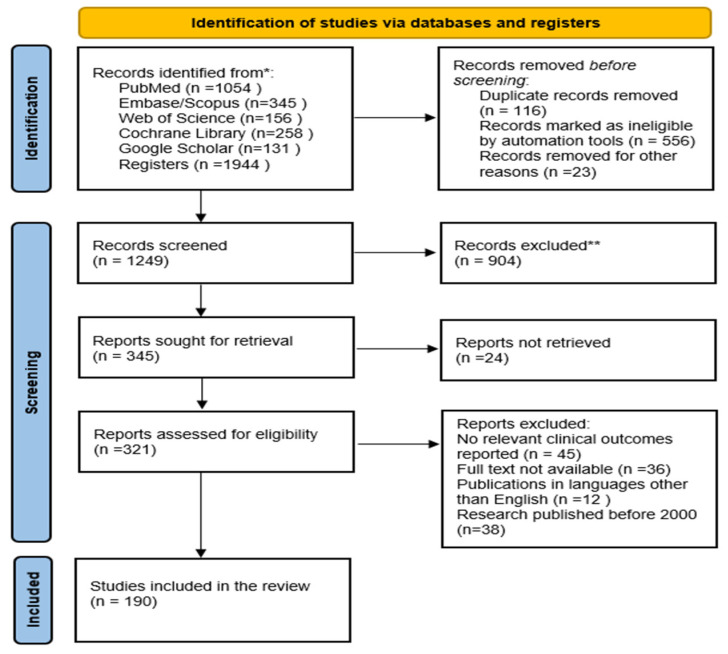
PRISMA flow diagram for identification of studies retrieved from databases.

The literature search identified a total of 1944 articles across six major electronic databases: PubMed (n = 1054), Embase/Scopus (n = 345), Web of Science (n = 156), Cochrane Library (n = 258), and Google Scholar (n = 131). Following the screening of titles and abstracts, 1623 articles were excluded and the full texts of the remaining 321 articles were retrieved. The subsequent analysis for adherence to the inclusion criteria identified 190 studies as eligible. The included studies were selected according to the process illustrated in the PRISMA flow diagram (Figure 4).

## 3. Results and Discussion

This review aims to systematically assess the roles of micronutrient and nutraceutical supplementation approaches in wound healing. Specifically, it seeks to analyze the effects of nutritional supplementation on tissue regeneration, inflammation reduction, and healing time, with a particular focus on diabetic wounds.

A wide range of nutrients—including vitamins (complex A, B, C, D, and E), minerals (zinc, iron, calcium, copper, magnesium, and selenium), and amino acids (arginine and glutamine)—are essential for various aspects of the wound healing process. This underlines the importance of a balanced nutritional approach to wound management [173]. Some studies have suggested that vitamin E may be more effective in improving cell viability, proliferation, and migration, particularly when combined with vitamin C. Vitamin A also functions synergistically with other nutrients to promote wound healing; for example, vitamin A improves the release of cytokines during the inflammatory phase, whereas vitamin C improves neutrophil migration and lymphocyte activation. During the proliferative phase, vitamin C stimulates collagen synthesis, whereas vitamin A promotes epithelial cell differentiation [174]. Zinc is essential for DNA and protein synthesis as well as cell division, thereby complementing the functions of vitamin A in terms of cell proliferation and immunomodulation [175]. The application of topical vitamin K in conjunction with retinol has been found to reduce the duration of laser-induced purpura [55].

A notable example of synergy is the interplay between vitamins D and K in bone and cardiovascular health. Vitamin D aids in the production of vitamin K-dependent proteins, which require vitamin K-mediated carboxylation to function effectively. This synergism is supported by animal and human studies, showing that optimal concentrations of both vitamins are beneficial for bone strength and cardiovascular health. Jointly supplementing vitamins D and K has shown enhanced benefits when compared with supplementation with the individual vitamins [176].

Nutritional biochemistry highlights the importance of vitamin D in facilitating the absorption and assimilation of essential minerals such as calcium and magnesium; however, it can also enhance the uptake of toxic elements if mineral sufficiency is not maintained. Hence, ensuring an adequate balance of essential minerals alongside vitamin D is crucial for preventing the absorption of harmful elements [29].

Minerals such as calcium, magnesium, and phosphate are vital for bone health and other physiological functions. Deficiencies in these minerals can lead to osteoporosis and other health issues, while excessive intake may result in adverse health effects such as hypercalcemia or kidney stones. Maintaining adequate mineral levels is essential for preventing disorders related to both deficiencies and toxicity [177]. Table 2 outlines the physiological and toxic doses of the most important micronutrients and nutraceuticals involved in wound healing, along with the consequences of their toxicity.

The combination of probiotics with B vitamins has shown notable effects on nutrient absorption and gut health. Research has indicated that probiotics can enhance the absorption of vitamins such as B1, B3, B5, and B12 significantly, with improvements ranging from 14.5% to 71.2%. This interaction occurs primarily through gut microbiota-mediated mechanisms, rather than direct vitamin production by probiotics. Such combinations have also been found to alleviate colon damage and modulate the gut microbiota, leading to a higher abundance of beneficial bacteria such as Akkermansia [178].

Angiogenesis plays a crucial role in wound healing, enabling the delivery of oxygen and nutrients to healing tissues. While controlled angiogenesis is essential for proper wound repair, the excessive growth of blood vessels can lead to delayed wound closure, the remodeling of affected tissues, or other complications during the healing process. Angiogenesis in wound healing can be divided into two main phases: an initial pro-angiogenic phase involving the excessive formation of poorly differentiated blood vessels, followed by an anti-angiogenic phase in which the vascular network matures and the number of vessels is reduced [179]. The inadequate supply of nutrients and/or oxygen during the healing process can lead to delayed wound closure or chronic ulcer formation. Balancing pro- and anti-angiogenic factors, as well as considering the timing of angiogenic processes, is crucial for optimal wound healing and tissue remodeling [180].

Polyphenols promote angiogenesis through the improvement of antioxidant defenses, thus reducing oxidative stress and modulating inflammatory responses. They stimulate the production of pro-angiogenic factors such as the vascular endothelial growth factor (VEGF), placental growth factor (PlGF), and hypoxia-induced factor 1 alpha (HIF-1α) [181]. These factors are crucial for initiating and supporting the formation of new blood vessels in wound areas. Each type of polyphenol—including proanthocyanidins—may offer unique pathways and benefits in the wound healing process [182]. The synergistic effects of micronutrients and nutraceuticals are summarized in Table 3. Thus, further research will be crucial for optimizing their use in clinical applications.

Chronic wounds—such as diabetic foot ulcers and venous leg ulcers—often fail to heal on time due to persistent inflammation, inadequate nutrition, and infections. Furthermore, factors such as the inappropriate use of antibiotics, obesity, and existing vascular or neuropathic complications significantly predict the risk of severe complications. Poor glycemic control—as determined by high levels of glycated hemoglobin—is a critical risk factor for chronic wounds [183,184]. Leucine, isoleucine, and valine regulate protein synthesis via the mTOR signaling pathway. In particular, leucine promotes the growth and repair of tissues by enhancing the initiation of translation and anabolic responses in the muscles and skin. Cysteine acts as a rate-limiting agent in the synthesis of glutathione, which plays a protective role in oxidative stress environments such as those commonly observed in chronic wounds [185]. Nutraceuticals, micronutrients, and probiotics have been highlighted for their health-promoting properties and potential to manage chronic conditions [186,187]. Such an integrated approach could potentially transform chronic wound management, reduce morbidity, and promote recovery through enhanced nutritional support [188].

Disseminating knowledge to patients, their families, and healthcare providers about recent research advancements, such as the impact of nutrients on wound healing and the management of hypertrophic scars, can aid in the development of more effective treatment strategies and improve patient outcomes [189,190].

The findings are anticipated to contribute to the formulation of evidence-based recommendations for the integration of nutritional strategies into wound care protocols. The integration of essential vitamins, minerals, and nutraceuticals in the therapeutic management of patients with chronic, treatment-resistant wounds warrants consideration. Understanding the synergistic effects of nutritional factors is important for developing approaches for supplementation with micronutrients and nutraceuticals, particularly in a manner dependent on the stage of wound healing.

Future research endeavors should focus on optimizing formulation protocols to enhance the clinical applicability of micronutrient and nutraceutical treatments intended for wound management, as well as determining therapeutic dosages and any associated toxicities.

## 4. Conclusions

The interactions between nutrients create a complex web of effects that influence multiple stages of wound healing. While vitamin A primarily affects epithelial regeneration and extracellular matrix production, its synergy with vitamins C and B, zinc, and other nutrients can improve overall wound healing outcomes. B-complex vitamins are essential for cellular metabolism and energy production, and have been associated with enhanced healing in burn wounds.

The inadequate intake or deficiency of these micronutrients can significantly affect the wound healing process, leading to delayed healing, an increased risk of infection, and poor recovery outcomes. Therefore, ensuring a proper diet that is rich in vitamins and minerals is vital for optimal wound healing outcomes, particularly in individuals who are at risk of deficiencies due to chronic diseases, malnutrition, or post-surgical recovery. Supplementation can be considered in specific cases where deficiencies are identified, but should be performed under medical supervision.

Probiotics have been shown to positively influence skin health through promoting the proliferation of beneficial bacteria, suppressing pathogenic bacteria, and generating antimicrobial compounds. As such, they can boost the immune system, improve skin barrier components, and modulate skin inflammation. Omega-3 fatty acids also modulate inflammatory pathways, influencing T-cell differentiation and gene expression. They affect membrane composition, eicosanoid biosynthesis, cell signaling cascades, and gene expression in almost every cell in the body. In the field of wound healing, supplementation with eicosapentaenoic acid (EPA) has been correlated with elevated concentrations of interleukin-10 (IL-10) in tissues during the early phases of the healing process. Arginine and glutamine are integral for the modulation of immune responses during wound healing, functioning as “immunonutrients” that exert a substantial influence on various components of the immune system and tissue repair mechanisms. Arginine plays a crucial role in protein synthesis, cell signaling (via nitric oxide production), and cell proliferation (through its conversion to ornithine and polyamines). These functions make arginine an indispensable substrate for wound healing.

The effectiveness of polyphenols in wound healing does not depend solely on their antioxidant properties; they also exhibit anti-inflammatory and antimicrobial properties, which are crucial for the management of hard-to-heal wounds, especially in diabetic and elderly populations. Flavonoids exhibit powerful antioxidant properties that significantly contribute to the wound healing process. These compounds act as free radical scavengers and metal chelators and reduce oxidative stress, which is crucial for promoting tissue repair and regeneration. Their antioxidant activities help to protect cells from oxidative damage, thus supporting the healing process. Flavonoids can also chelate metal ions, which are involved in oxidative reactions, inhibit lipid peroxidation during wound healing by directly removing reactive oxygen species (ROS), enhance endogenous antioxidants, and promote synergistic interactions with other antioxidants. These mechanisms contribute to their potential as therapeutic agents for wound management, especially in diabetic conditions where oxidative stress is high.

Natural products containing saponins, essential oils, flavonoids, and phenolic compounds have also demonstrated potential for wound healing due to their anti-inflammatory, antioxidant, antibacterial, and pro-collagen synthesis properties. The integration of micronutrients and nutraceuticals into wound care protocols offers promising opportunities for the enhancement of therapeutic strategies, particularly in cases where conventional methods may prove insufficient. This highlights the importance of implementing a balanced nutritional approach in the context of wound management, particularly considering the interactions between various nutrients to facilitate optimal healing.

Significant variability was noted in study designs, sample sizes, intervention dosages, and outcome measures. This evidence underscores the necessity for further well-designed clinical trials to determine optimal dosages and combinations for specific wound types across diverse patient populations.

## Figures and Tables

**Table 1:** Roles of nutraceuticals in wound healing and their underlying mechanisms.

Nutraceuticals	Role in Wound Healing	Mechanisms of Action
**Polyphenols and flavonoids**	Supports vascular regeneration and prevents chronic inflammation	Inhibits the NF-κB pathway, pro-motes angiogenesis, and cell prolif-eration
**Saponins**	Anti-inflammatory, antioxidant, antibacterial, and pro-collagen syn-thesis properties	Exerts anti-inflammatory effects by regulating the NF-κB and MAPK pathways
**Arginine and glutamine**	Stimulates collagen formation and immune defense	Boosts nitric oxide synthesis, en-hances fibroblast activity, and im-proves collagen synthesis
**Omega-3 Fatty Acids**	Promotes cell migration and tissue regeneration	Suppress pro-inflammatory cyto-kines, activate high-conductance voltage-activated (BK) calcium (Ca2+) and potassium (K+) channels, which contribute to vasodilation
**Probiotics**	Supports systemic immune balance via gut-skin axis, improves skin barrier, and reduces infection risk	Reduces pro-inflammatory cytokine levels, stimulates local cell growth and repair, and protects against pathogens

**Table 2:** The physiological and toxic doses of the most important micronutrients and nutraceuticals involved in wound healing, along with the consequences of their toxicity.

**Compounds**	Physiological Doses	Toxic Doses	Toxicity Effects
**Vitamin A**	700–900 µg/day	>3000 µg/day	Hepatotoxicity, headaches, osteoporosis
**Vitamin C**	75–90 mg/day	>2000 mg/day	Gastrointestinal upset and kidney stones
**Vitamin B1**	1.1–1.2 mg/day	No UL	Generally safe
**Vitamin B6**	1.3–2.0 mg/day	>100 mg/day	Neuropathic symptoms, abdominal pain
**Vitamin B12**	2.4 -3 µg/day	No UL	Generally safe
**Vitamin E**	15 mg/day	>1000 mg/day	Bleeding risk, amplified risks of cardiovas-cular events, and fatigue
**Vitamin D**	15–20 µg/day (600–800 IU)	>100 µg/day (>4000 IU)	Hypercalcemia, nausea, vomiting, polyuria, polydipsia, dehydration, confusion, apathy
**Vitamin K**	50–200 µg/day	Low toxicity	Generally safe
**Zinc**	8–11 mg/day	>40 mg/day	Copper deficiency, anemia, leukopenia, nausea, and memory impairment
**Selenium**	55 µg/day	>400 µg/day	Hair loss, nail brittleness, gastrointestinal disturbances
**Magnesium**	310–420 mg/day	>350 mg/day	Diarrhea, hypotension
**Iron**	8–18 mg/day	>45 mg/day	Gastrointestinal distress, hemosiderosis
**Omega-3 (EPA/DHA)**	250–500 mg/day	>3000 mg/day	Bleeding risk, immune suppression
**Arginine**	6–18 g/day	>30 g/day	Gastrointestinal upset, hypotension, and viral reactivation risk
**Glutamine**	5–10 g/day-30 g/day	>0.75 g/kg/day	Elevated ammonia, Gastrointestinal dis-tress, liver strain
**Resveratrol**	100–500 mg/day	>1000 mg/day	Unknown long-term toxicity
**Curcumin**	500–2000 mg/day	>8000 mg/day	Gastrointestinal distress, liver enzyme ele-vation
**Proanthocyanidis**	50–300 mg/day	Generally safe up to 1000 mg/day	Very high doses may cause gastrointestinal disturbances or interfere with nutrient ab-sorption
**Quercetin**	500–1000 mg/day	>1000 mg/day	Headaches, kidney stress (high doses)
**Fisetin**	100–200 mg/day	>1000 mg/day	Possible liver damage at high doses
**Saponins**	Varies by source; ~10–100 mg/day	>10 g/kg	Gastrointestinal distress, diarrhea, and possible liver/kidney effects

**Table 3:** Nutritional synergies for wound healing and tissue regeneration.

Category	Synergistic Component	Role in Wound Healing and Tissue Regeneration
**Colagenogenesis**	Vitamin C Vitamin A Zinc	Stimulates collagen synthesis and tis-sue remodeling
**Regenerate Tissue**	Arginine Glutamine Vitamin B complex	Promotes cell proliferation and tissue restoration
**Local immunity**	Probiotics Vitamin D Zinc Selenium	Improves immune response and pro-tects the epithelial barrier
**Antioxidant, Anti-inflammatory**	Omega 3 Curcumin Vitamin E	Reduces inflammation and oxidative stress
**Angiogenesis**	Vitamin D Arginine Polyphenols	Stimulates the formation of blood vessels in the affected tissues

## Data Availability

Not applicable.
